# Society of Surgery—Meeting of August 5, 1846

**Published:** 1846-10

**Authors:** 


### *Society of Surgery—Meeting of August 5, 1846.*

CATARACT.—Professor Berard presented to the meeting a crystalline lens of a stony consistency, which he had extracted under the following circumstances:--A lady, aged 35, was since her birth blind in the left eye, and, fifteen years ago, on examination, the blindness was found to depend on opacity of the lens. Towards the end of the month of June last, without any apparent cause, the patient experienced a sudden pain in the head, and, on looking into the eyes, the lens was seen in the anterior chamber; a violent ophthalmia having supervened, was treated without success by antiphlogistic measures and purgatives; the removal of the lens was accordingly resolved upon. The division of the cornea was performed with a great deal of pain, and the lens was extracted; a broad strip of adhesive plaster was placed over the superior eyelid, and the inflammation gradually subsided after the operation, although it was followed by excessive suffering. Vision has not been restored.

M. Robert had seen similar cases, and would have followed the plan indicated by Sanson, viz., dividing the cornea in its upper part.

M. Nélaton attributed the displacement of the lens to the great dilatation of the pupil; he had observed that in these cases the passage of the lens into the anterior chamber usually took place during the night.

ANEURISM.--M. Malgaigne related the case of a child who presented on one of the anterior branches of the temporal artery two small aneurismal tumours of the size of large peas, which he at first intended to operate upon by M. Petrequin's method of galvano-puncture. But, being unable to procure a proper battery, he merely passed pins through the tumours, and threw over each a twisted suture, which he left for twelve days in situ; at the end of that period the cure was complete.

Hydatic Tumour of the Womb.—M. Malaigne then laid on the table a uterus containing an enormous hydatic cyst. The following is the history of the case:—

E. S., aged twenty-seven, had always been a woman of delicate health. Almost every day she was affected with some sickness, and threw up bile and food; at eighteen she was married, and first menstruated only at the age of nineteen. Her first pregnancy, which took place soon after, was not attended with any peculiar symptoms, and she gave birth to a child who lived nine months, and died from convulsions. Twenty months ago she again became pregnant, and the child lived but one month; in both pregnancies the uterus was extremely distended, and the quantity of the amniotic fluid appeared unusually considerable. Since the month of May last the menses had not been observed, and, from the middle of June, the abdomen began to swell, and had attained at the end of July the size of an eight months' pregnancy. She was admitted into the hospital on the 24th of July, and complained of slight hemorrhage and continual vomiting; she also suffered from cramps in the legs, headache, giddiness, and loss of appetite. Examination per vaginam showed the os-tincæ to be extremely high, but healthy. Eight ounces of blood were taken from the arm, and the patient declared herself much relieved, the uterine hemorrhage having stopped. During the night of July 26th, the discharge of blood re-appeared, and with such violence that in the space of a quarter of an hour the two mattresses and the bed-clothes were completely soaked through. Notwithstanding cold lotions, and the application of a plug, the hemorrhage returned, and the patient died in the morning.

Dissection showed that the distended uterus occupied the whole abdominal cavity, and the womb, removed from the body, shot forth through the os-tincæ a considerable amount of arterial blood. The transversal circumference of the viscus measured sixty centimetres (twenty-one inches), and the perpendicular circumference sixty-six (twenty-five inches). On opening the uterus it was found to contain, not a fœtus, but an immense number of transparent vesicular bodies of the size of peas, attached to each other in bunches; no trace whatever of a placenta was detected. This mass did not adhere to the uterine walls; and in the fundus was noticed a surface larger than the palm of the hand, and deprived of that polished appearance observable in the other parts. The thickness of the parietes of the womb was from four to six lines.

M. Gosselin had no doubt that it was a case of diseased placenta. The special tissue was not, it is true, present; but similar instances have been repeatedly met with in which the placenta is positively visible, enclosing the hydatids, the formation of which causes the embryo to disappear.

M. Monod regretted that no effort had been made to arrest the fatal hemorrhage. He was of opinion that compression of the aorta ought to have been tried.

Albuminous Nephritis in Pregnant Women.—This subject has been the object of the particular study of Dr. Cahen, late *interne* of the Parisian hospitals. The following is the result of his experience: In pregnant, but healthy, women the urine is never albuminous; it should be tested with the greatest care where the patient complains of unusual lumbar pains, &c., when the lower extremities or the face are the seat of any œdema. Pregnancy seems to favour the production of albuminuria; and this complication is a frequent cause of premature delivery, and of convulsions. Indeed Dr. Cahen asserts that he never met, in any case, with albuminous urine during pregnancy, without the discovery being followed by one or the other of the accidents above mentioned. After confinement the urine generally returns rapidly to a healthy condition; the anatomical alterations observed, after death from convulsions, all belong to those attributed to granular kidney. The best treatment consists in venesection.—*Lon. Med. Times.*

